# Food-Borne Viruses in Shellfish: Investigation on Norovirus and HAV Presence in Apulia (SE Italy)

**DOI:** 10.1007/s12560-016-9273-1

**Published:** 2016-12-10

**Authors:** G. La Bella, V. Martella, M. G. Basanisi, G. Nobili, V. Terio, G. La Salandra

**Affiliations:** 1 Istituto Zooprofilattico Sperimentale della Puglia e della Basilicata, Foggia, Italy; 2 Dipartimento di Medicina Veterinaria, Università degli Studi di Bari “Aldo Moro”, Valenzano (BA), Italy

**Keywords:** Norovirus, Hepatitis A, Real-time PCR, Genotyping, Shellfish

## Abstract

Shellfish are an important vehicle for transmission of food-borne pathogens including norovirus (NoV) and hepatitis A virus (HAV). The risks related with consumption of shellfish are greater if these products are eaten raw or slightly cooked. As molluscs are filter-feeding organisms, they are able to concentrate pathogens dispersed in the water. Data on shellfish viral contamination are therefore useful to obtain a background information on the presence of contamination in the environment, chiefly in shellfish production areas and to generate a picture of the epidemiology of viral pathogens in local populations. From January 2013 to July 2015, 253 samples of bivalve molluscs collected in harvesting areas from a large coastal tract (860 km) of Southern Italy were screened for HAV and NoV of genogroups GI and GII, using real-time reverse transcription qualitative PCR. The RNA of HAV was not detected in any of the analyzed samples. In contrast, the RNA of NoV was identified in 14.2% of the samples with a higher prevalence of NoVs of genogroup GII (12.2%) than genogroup GI (1.6%). Upon sequence analysis of a short diagnostic region located in capsid region, the NoV strains were characterized as GII.2, GII.4 Sydney 2012, GII.6, GII.13, GI.4, and GI.6, all which were circulating in local populations in the same time span. These data confirm that consumption of mussels can expose consumers to relevant risks of infection. Also, matching between the NoV genotypes circulating in local population and detected in molluscs confirms the diffusion in the environment of NoVs.

## Introduction

Food-borne viruses are an important and emerging problem for food safety and public health. According to a report by EFSA (2015), in 2014 viruses were, for the first time, the most commonly detected (20.4%) causative agent in food-borne outbreaks. Filter-feeding shellfish is an important vehicle for transmission of food pathogens including enteric viruses such as norovirus (NoV) and hepatitis A virus (HAV) when grown in sewage-polluted water (Lees 2000), since these molluscs are able to accumulate and concentrate pathogens present in the water (Le Guyader et al. 2000). The risks related with consumption of shellfish are greater when these products are eaten raw or slightly cooked, as in some European countries, including the southern regions of Italy. This appears to affect heavily the epidemiology in local populations of some human infectious diseases (Terio et al. 2010). The European Regulation 2073/2005, and subsequent amendments, defines food safety criteria of shellfish only on the basis of bacterial indicators (e.g., *Salmonella* and *Escherichia coli*) that may not be correlated with the presence of viruses (Goyal et al. 1979; Croci et al. 2000; Koopmans and Duizer 2004). Enteric viruses are more resistant to inactivation in water sources and are removed slowly, or not at all, from bivalves by depuration process (De Medici et al. 2001; Croci et al. 2007). Gathering information on virus contamination in shellfish has therefore become increasingly important in countries with relevant production. Italy is the third main European producer of bivalve molluscs, with an average of 100,000 tons per year (Bronzi et al. 2011) and a large part of this production is concentrated in the Apulia region (MIPAAF 2014).

HAV is a non-enveloped 7.5-kb positive-stranded RNA virus of the family *Picornaviridae* genus *Hepatovirus* (Manso and Romalde 2013). HAV is responsible for human acute viral hepatitis. Only one serotype of HAV has been identified worldwide. Genetic heterogeneity of HAV allows classifying HAV strains into seven different genotypes, named I–VII. Genotypes I and III have been further divided into sub-genotypes A and B (Robertson et al. 1992).

NoV, “Norwalk-like virus,” family *Caliciviridae*, is considered the main cause of acute gastroenteritis in children and adults (Koopmans 2008). NoVs are a non-enveloped viruses with a 7.5–7.7-kb positive-sense single-stranded RNA genome containing three open reading frames (Vinjé 2015). NoVs are classified genetically into six genogroups, GI–GVI (Green 2013), of which GI, GII, and GIV have been identified in humans. Each genogroup is further classified into several genotypes on the basis of the capsid gene sequences (Kroneman et al. 2013; Zheng et al. 2006).

Human NoV and HAV grow very poorly or they do not grow at all in vitro, and their detection in food matrices relies on molecular techniques. A standardized method for virus detection and quantification in food, including shellfish, is now available (ISO/TS 15216 part 1 and part 2, International Organization for Standardization 2013a, b).

The aim of this study was to collect data on the prevalence of HAV and NoV in shellfish from harvesting areas of Apulia region (Southern Italy) with a large coastal tract of 860 km, where a large part of Italian production is concentrated. Sequence analysis of the detected strains was also carried out to contribute to epidemiological studies on these food-borne viruses in Italy, as well as the associated health risk for consumers.

## Materials and Methods

### Sample Collection and Processing

The current European legislation (Anonymous 2004) classified the molluscan shellfish harvesting areas into A, B, or C category on the basis of *E. coli* levels as follows: A (<230 *E. coli* cfu/100 g shellfish), B (<4600 *E. coli* cfu/100 g shellfish), and C (4600–46,000 *E*. *coli* cfu/100 g shellfish). The total coastline of the Italian peninsula is about 7500 km, and the Apulia region (South East of Italy) alone has nearly 800 km (13.3%) of the Italian coastline. In the Apulia region, there are nine harvesting areas of which eight are classified as A and one is classified as B, by the official authority of Apulian Regional Government.

From January 2013 to July 2015, a total of 253 samples of different shellfish species from harvesting areas of the Apulia region, 219 from area A and 34 from area B, were analyzed. The bivalve species analyzed included mussels (*Mytilus galloprovincialis*, *n* = 181), clams (*Venus gallina*, *n* = 34), oysters (*Crassostrea gigas*, *n* = 22), and other species (*Modiolus barbatus*, *Acanthocardia tuberculata* and *Solen marginatus*, *n* = 16). All the collected samples were transferred and preserved, until analysis, at 4 °C and were subjected to viral analysis for determination of HAV and NoV GI and GII using the molecular method described by international standard ISO/TS 15216-2 (International Organization for Standardization 2013b).

Depending on species size, 15–60 individuals of each sample were randomly selected for the analysis and digestive tissue was dissected, cleaned, and finely chopped with a sterile razor. Aliquots of 2.0 g, spiked with 10 μl of process control (titrated suspension of Mengovirus strain MC_0_ supplied by Istituto Superiore di Sanità, Rome, Italy), were treated for digestion with 2 ml of proteinase K (0.1 mg/ml) at 37 °C for 60 min with shaking, and then placed at 60 °C for 15 min to produce inactivation of the enzyme. Finally, the samples were centrifuged at 3000×g for 5 min, and the supernatant was collected and volume was measured (range from 2.3 to 3.0 ml). Nucleic acid extraction and purification were performed using the Nuclisens extraction kit (BioMerieux, Paris, France) according to the manufacturer’s instructions, and the eluted RNA (100 μl) was stored at −80 °C until real-time RT-PCR analysis.

### Real-Time RT-PCR

The real-time RT-PCR for HAV and NoV detection was carried out on a 7500 Fast Real-Time PCR system (Applied Biosystems, Foster City, California, US) using amplification conditions, primers, probes, and reagents (RNA UltraSense™ One-Step Quantitative RT-PCR System, Life Technologies, Carlsbad, California, US) reported in ISO/TS 15216-2:2013 (International Organization for Standardization 2013b). Primers and probes sequences are listed in Table 1. Undiluted and 1:10 diluted samples were tested. The presence of PCR inhibitors was evaluated by testing samples along with an external control RNA (EC-RNA, approximately 10^4^ copies of target sequence) and amplification efficiency was calculated by comparing the Ct value of EC-RNA alone (E = 2^−ΔCt^). Results from undiluted samples were considered acceptable if amplification efficiency was ≥50%; otherwise, only results from dilution 1:10 were considered. In each run, two negative controls (molecular grade water) and a positive control (the same EC-RNA as above) were added. The efficiency of the extraction procedure was evaluated through the recovery of the process control, comparing the Ct values obtained for Mengovirus on shellfish samples extracts to the viral stock, taking into account the dilution factor due to the extraction procedure and the aliquot of sample subjected to analysis. Recovery was considered acceptable if ≥1%; samples failing to reach this criterion were re-extracted.

**Table 1:** Primers and probes for HAV, NoV GI, NoV GII, and Mengovirus detection

Virus	Primer	Sequence	References
Hepatitis A virus	HAV68 (F)	5′-TCACCGCCGTTTGCCTAG	Costafreda et al. (2006)
	HAV240 (R)	5′-GGAGAGCCCTGGAAGAAAG-3′	Costafreda et al. (2006)
	HAV150 (P)	5′-FAM-CCTGAACCTGCAGGAATTAA-MGBNFQ-3′	Costafreda et al. (2006)
NoV GI	QNIF4 (F)	5′-CGCTGGATGCGNTTCCAT-3′	Da Silva et al. (2007)
	NV1LCR (R)	5′-CCTTAGACGCCATCATCATTTAC-3′	Svraka et al. (2007)
	NVGG1p (P)	5′-FAM-TGGACAGGAGAYCGCRATCT-TAMRA-3′	Svraka et al. (2007)
NoV GII	QNIF2 (F)	5′-ATGTTCAGRTGGATGAGRTTCTCWGA-3′	Loisy et al. (2005)
	COG2R (R)	5′-TCGACGCCATCTTCATTCACA-3′	Kageyama et al. (2003)
	QNIFs (P)	5′-FAM-AGCACGTGGGAGGGCGATCG-TAMRA-3′	Loisy et al. (2005)
Mengovirus	Mengo110 (F)	5′-GCGGGTCCTGCCGAAAGT-3′	Pintó et al. (2009)
	Mengo209 (R)	5′-GAAGTAACATATAGACAGACGCACAC-3′	Pintó et al. (2009)
	Mengo147 (P)	5′-FAM-ATCACATTACTGGCCGAAGC-MGBNFQ-3′	Pintó et al. (2009)

*F* forward/sense, *R* reverse/antisense, *P* probe, FAM 6-carboxyfluorescein (reporter dye), *MGBNFQ* minor groove binder/non-fluorescent quencher, TAMRA 6-carboxy-tetramethylrhodamine (quencher dye)

### HAV and NoV Genotyping

The samples that tested positive for either HAV or NoV in real-time RT-PCR were subjected to further investigations for the identification of viral genotype.

For genotyping of HAV, a nested RT-PCR assay was used, based on gene-specific primers (dkA24−dkA25) targeting the VP1/2A junction region (Kingsley and Richards 2001). A 200-bp fragment of viral RNA was amplified using the Hot Star Taq Master mix kit (Qiagen, Milan, Italy). First-round RT-PCR was performed using superscript II one step (Invitrogen, Paisley, UK) in order to amplify a 267-bp fragment (Robertson et al. 1992).

For NoV, a hemi-nested RT-PCR protocol was used, targeting a highly conserved region in the diagnostic region C (ORF2) located on the capsid protein VP1, with minor modifications (Kojima et al. 2002; Nishida et al. 2003). Primers for the first PCR were as follows: 5′-CGY TGG ATG CGN TTY CAT GA-3′ (COG1F; sense), 5′-CCA ACC CAR CCA TTR TAC A-3′ (G1-SKR; antisense), 5′-CAR GAR BCN ATG TTY AGR TGG ATG AG-3′ (COG2F; sense), and 5′-CCR CCN GCA TRH CCR TTR TAC AT-3 (G2-SKR; antisense). Primers for the hemi-nested PCR were 5′-CTG CCC GAA TTY GTA AAT GA-3 (G1-SKF; sense), 5′-CCA ACC CAR CCA TTR TAC A-3′ (G1-SKR; antisense), 5′-CNT GGG AGG GCG ATC GCA A-3′ (G2-SKF; sense), and 5′–CCR CCN GCA TRH CCR TTR TAC AT-3′ (G2-SKR; antisense). All the PCRs were performed in a GenAmp PCR System 2007 thermal cycler (Applied Biosystem) using the superscript II one step (Invitrogen, Paisley, UK) in the first-round PCR and the Hot Star Taq Master mix kit (Qiagen, Milan, Italy) in the second-round PCR.

### Sequence Analysis

The amplicons were excised from the gel and purified by the commercial extraction kit QIAquick Gel Extraction Kit (Qiagen, Milan, Italy). Sequencing was carried out using BigDye Terminator Cycle chemistry (Applied Biosystems, Foster City, California, US). Raw sequences were edited using the Geneious software version 9.1.6 (Biomatters Ltd, New Zealand) and compared with reference strains retrieved from GenBank.

NoV genotypes were assigned based on clustering with reference strains from the sequence database of the European Network NoroNet using the Norovirus Typing Tool database (http://www.rivm.nl/mpf/norovirus/typingtool) (Kroneman et al. 2011).

Furthermore, the obtained sequences were analyzed using an Italian database maintained by the Italian study group for enteric viruses (ISGEV; http://isgev.net) that monitors the epidemiology of enteric viruses in children through hospital-based surveillance.

For phylogenetic analysis, the sequence alignments were generated with ClustalW and analyzed with Geneious software package. The phylogenetic trees were generated using the neighbor-joining method, with the HKY851 correction method and with bootstrap analysis over 1000 replicates.

### Statistical Analysis

An analysis of two-by-two contingence table with positive and negative results from harvesting areas A and B, using the Fisher’s exact test, was performed. Analysis and evaluation of significance (*p* value) were performed using the GraphPad Software QuickCalcs (http://www.graphpad.com/quickcalcs/contingency1/).

## Results

**Fig. 1:**
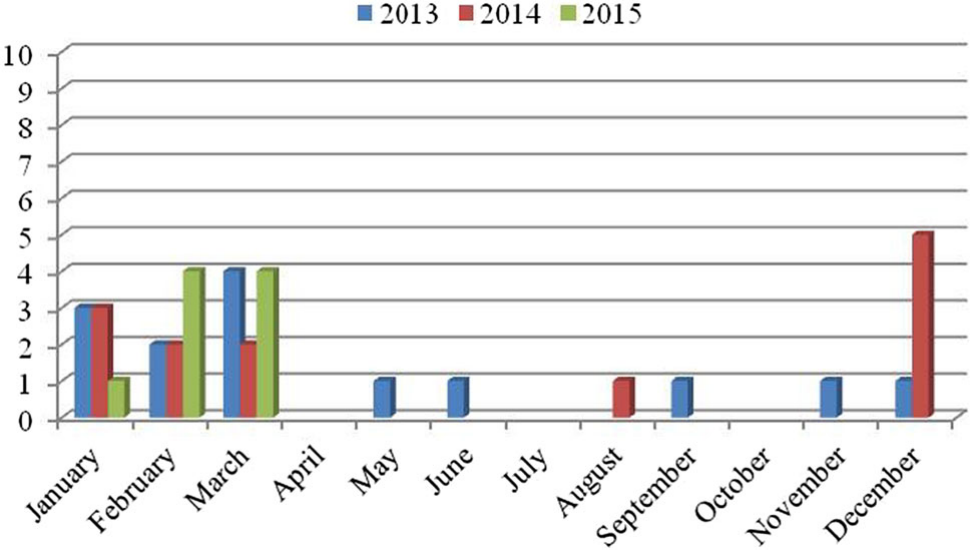
Variations of positive samples during the period of investigation. Although the presence of NoV was detected in all the months, the frequency of NoV detection markedly increased from December to March for all the 3 years

**Fig. 2:**
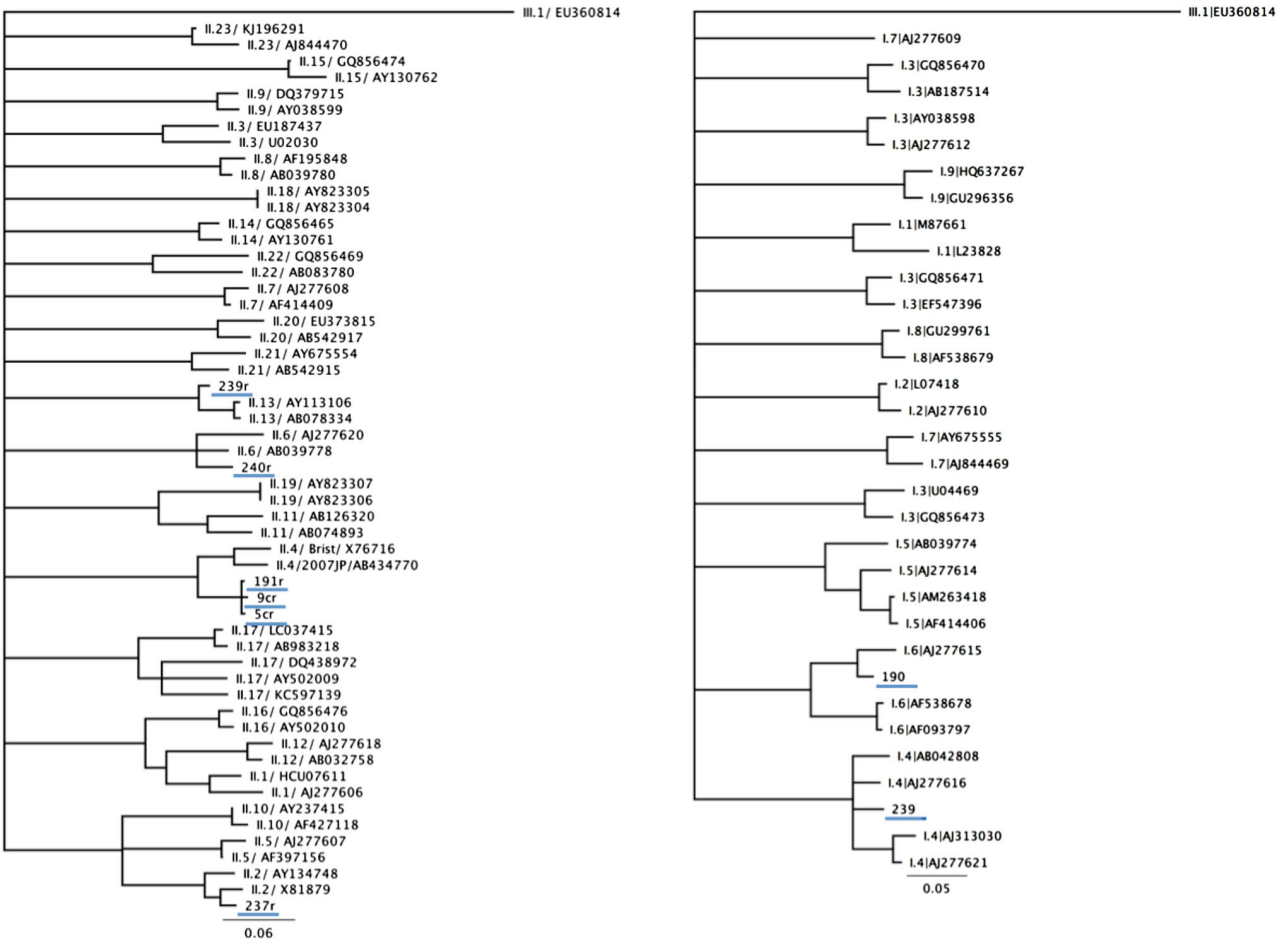
Phylogenetic trees for the NoV GI (on the *right*) and NoV GII (on the *left*) sequences detected in shellfish based on the partial ORF2 sequences of region C. The alignment of the sequences was carried out by ClustalW and the phylogenetic trees were generated using the neighbor-joining method, with the HKY851 method and with bootstrap analysis over 1000 replicates

Of the 253 analyzed samples, the nucleic acid of NoV was detected in 36 samples (14.2%). All the 253 samples were negative for HAV in real-time RT-PCR. The results are shown in Table 2. Out of 36 NoV-positive samples, 4 were NoV GI (11.1%), 31 were NoV GII (86.1%), and only one sample contained both genogroups GI and GII (2.8%). Two hundred and nineteen samples were collected from harvesting areas classified as zone A. Of these samples, 23 (10.5%) were positive for the presence of NoV. Four (17.4%, 4/23) samples were positive for GI, 18 (78.6%, 18/23) were positive for GII, and one was positive for both GI and GII. The other 34 samples analyzed were collected from harvesting areas classified as zone B. NoV was detected in 13 (38.2%) of them, all positive for NoV GII, with an extremely statistically significant association (*p* = 0.0001). With regard to the distribution of the contamination during the period of investigation, the presence of NoV was detected in all the months. However, the frequency of NoV detection markedly increased from December to March (Fig. 1). The differences among contamination frequencies of the species are reported in Table 2. Genotyping of NoV based on the capsid region C allowed characterizing 22.2% (8/36) of the samples (Fig. 2). Six samples were GII strains and two were GI strains. Of the six GII NoV-positive samples, one was characterized as GII.2 (#237), three as GII.4 variant Sydney 2012 (#5c, #191, #9c), one as GII.6 (#240), and one as GII.13 (#239). Of the two GI NoV strains, one sample was characterized as GI.4 (#239) and the other one as GI.6 (#190). Sample #239 contained the RNA of two different NoV genotypes, GII.13 and GI.4. All the identified strains were obtained from samples collected between March 2013 and December 2014.

**Table 2:** Results for detection of HAV and NoV from each species

Species	No. of analyzed samples	Viral contamination				
		HAV (%)	NoV GI (%)	NoV GII (%)	NoV GI + GII (%)	Total (%)
*Mytilus galloprovincialis*	181	0	3	26	1	30 (16.6)
*Venus gallina*	34	0	1	1	0	2 (5.9)
*Ostrea spp.*	22	0	0	1	0	1 (4.5)
*Modiolus barbatus*	9	0	0	1	0	1 (11.1)
*Acanthocardia tuberculata*	6	0	0	1	0	1 (16.7)
*Solen marginatus*	1	0	0	1	0	1
Total	253	0	4 (1.6)	31 (12.2)	1 (0.4)	36 (14.2)

## Discussion

This study provides the findings of a surveillance work for viral enteric pathogens in harvesting shellfish production areas of Apulia region (SE Italy), with the application of the standardized protocol ISO/TS 15216-2 (International Organization for Standardization 2013b).

The results showed a clear picture of the situation of the region in the years 2013–2015. The absence of HAV in the analyzed samples confirms the low prevalence of this virus in Italian areas of production of shellfish, as reported in previous studies (Croci et al. 2007; Suffredini et al. 2008) and in similar studies conducted in Apulia region in the years 2009–2013 (Terio et al. 2014). This might be related to either the low circulation of HAV in the study period or to the negative trend of HAV infection in Italy in the last 10 years (SEIEVA 2014; Suffredini et al. 2014) and in Apulia region (Chironna et al. 2012). HAV has long been a serious public health problem in Europe and in particular in Apulia, where between 1996 and 1997 a large epidemic occurred. After this epidemic, the incidence of HAV has steadily declined since 2008 (Terio et al. 2014). One crucial point for HAV control in Apulia is due to the actual policy of universal vaccination of toddlers and adolescent (Chironna et al. 2012).

A different scenario was depicted for NoVs. The prevalence of NoV in shellfish revealed in our survey (14.23%) is similar to what observed in other studies (Terio et al. 2014, 2010; Croci et al. 2007; Le Guyader et al. 1998). Previous studies conducted in Italy at a regional level showed a high variability in the NoV contamination rates in filter-feeding shellfish in the last 10 years, from 4.1 (Pavoni et al. 2013) to 34.4% (Suffredini et al. 2011), 51.4 (Suffredini et al. 2012), 51.5 (Suffredini et al. 2014), and 57.7% (Pepe et al. 2012). The yearly variations in the contamination frequency of seafood products may indicate that the levels of virus dispersion and presence in the environment are not uniform. This may be related to the unequal distribution of NoV gastroenteritis in Europe (Kroneman et al. 2008; Pavoni et al. 2013) or may be accounted for by different contamination levels of the harvesting areas due to geographical factors (e.g., distance from the coast, from rivers, and so on).

With regard to the prevalence of the genogroups of NoV, the results confirm the higher circulation of NoV GII with respect to GI in shellfish (Suffredini et al. 2014; Pavoni et al. 2013; Terio et al. 2014; Manso and Romalde 2013). In fact, GII viruses are detected most frequently (89%), whereas GI viruses approximately cause 11% of the outbreaks (Vega et al. 2014). The causes of the high prevalence of GII NoVs are still unknown. Possible explanations include differences in biological properties such as virulence, routes of transmission, or stability of the virus in the environment (Croci et al. 2007). Other studies also supposed that the higher prevalence of NoV GII may be due to a different affinity to mussel tissues that may influence the ability to bioaccumulate in shellfish (Suffredini et al. 2011; Comelli et al. 2008). Also, the epidemiological pattern observed in shellfish could be a mere consequence of the higher prevalence of GII NoVs in human population (Manso and Romalde 2013). The predominance of GII NoV strains in human population seems to be accounted for by their ability to recognize a broader range of receptors, i.e., the histo-blood group antigens (HBGA) family (Lindesmith et al. 2012) than other NoVs.

Interestingly, a temporal pattern was noted in the NoV detection rates in shellfish. NoVs could be detected throughout the year, but the frequency was higher from December to March (Fig. 1). Many studies have reported a seasonal distribution of NoV detection in shellfish samples mainly in the cold months (Le Guyader et al. 2000; Lowther et al. 2012; Suffredini et al. 2012), thus suggesting seasonality for these virus infections (Croci et al. 2007). NoV illnesses related to shellfish consumption present a seasonal pattern, generally showing a peak incidence during the wintertime (Rippey 1994; Rohayem 2009). This seasonality could be attributed to several factors, including increased stability of viruses at low water temperature, reduced solar inactivation, and selective bioaccumulation of these pathogens by the shellfish (Suffredini et al. 2012). In addition, to the latitude (and their associated climatologic and oceanographic characteristic) (Polo et al. 2015), other factors could be a cause of seasonality, such as increases in tourism and/or consuming rates, seasonality. Nonetheless, atypical spring and summer peaks of NoV detection have been also reported (Lopman et al. 2004b; Polo et al. 2015). At the same time, it is well known that the activity of NoVs is subjected to seasonal fluctuations, with a winter peak of outbreaks in temperate climates (Mounts et al. 2000; Lopman et al. 2004a). This could result in a greater contamination of water and consequently of mussels.

The difference between the prevalences of positive samples from harvesting areas A and B (*p* = 0.0001) showed a correlation between NoV levels and classification of harvesting areas. These data are in accordance with previous studies, which confirmed such correlation, probably related to the higher impact of fecal contamination in class B areas. These findings underline the necessities of improving the management of pollution sources and of devising post-harvest treatments (Suffredini et al. 2014; Lowther et al. 2012).

Despite the high number of genotypes and genetic diversity of NoVs (Kroneman et al. 2013), viruses from a single genotype, GII.4, are responsible for the majority of the NoV outbreaks and sporadic cases of gastroenteritis worldwide (Siebenga et al. 2009; Bok et al. 2009), whereas GI strains are more often detected in food-borne and waterborne outbreaks (Vinjé 2015). GII.4 NoV strains continuously undergo genetic/antigenic diversification and periodically generate novel strains through accumulation of punctuate mutation or recombination (Green 2013). Since the mid-1990s, seven different GII.4 variants have successively emerged every 2–3 years, replacing previous dominant variants, and some of the GII.4 variants have been associated with global epidemics of gastroenteritis (Vinjé 2015). From the end of 2011, the variant GII.4 Sidney 2012 became predominant in Italy (Giammanco et al. 2013), mirroring the changes of NoV epidemiology observed on a global scale. However, between 2009 and 2013, several non-GII.4 strains (GII.12, GII.1, GI.6) have also co-circulated along with the predominant GII.4 viruses (Vinjé 2015). Interestingly, the NoV strains detected in shellfish in our survey appeared to reflect the circulation of NoV genotypes in local population. As reported in Table 3, all the GII strains identified in mussels were also reported in Apulia in the same time span during surveillance in hospitalized pediatric patients. In particular, the strains GII.4 Sydney 2012 have been the dominant variant in Italy and in Apulia since the 2012–2013 winter season (Medici et al. 2014). These findings seem to suggest that the NoV sequences generated from mussels faithfully reflect the epidemiological situation of the local territory.

**Table 3:** Chronology of the NoV-positive shellfish samples identified in Apulia and of the strains identified in hospital-based surveillance in local population in the same time span

# Sample	Date	Genotype	Date of detection by hospital-based surveillance
190	March 2013	GI.6	N.D.
191	March 2013	GII.4 Sydney 2012	November 2012
237	January 2014	GII.2	March 2013
239	January 2014	GII.13	January 2015
		GI.4	N.D.
240	January 2014	GII.6	November 2014
5c	December 2014	GII.4 Sydney 2012	November 2012
9c	December 2014	GII.4 Sydney 2012	November 2012

*N.D.* not detected

## Conclusion

The present survey provides data on the presence and diffusion of NoV and HAV in Apulia region between 2013 and 2015. The findings confirm that NoVs can be easily detected in mussels, thus confirming the potential role of this food for transmission of viral gastroenteritis. The data were obtained using standardized and internationally recognized methods in response to EFSA request for data on viral hazards in food. Continual surveillance for food-borne viral pathogens is necessary to estimate with more precision viral hazards. Also, the development and/or refinements of the diagnostic methods are pivotal, as novel viral strains can periodically emerge and challenge the diagnostics. These data will be useful for the optimization of monitoring strategies and for the definition of criteria for the presence of food-borne viruses in shellfish.
